# Thrombospondin-2 as a diagnostic biomarker for distal cholangiocarcinoma and pancreatic ductal adenocarcinoma

**DOI:** 10.1007/s12094-021-02685-8

**Published:** 2021-07-28

**Authors:** J. Byrling, K. S. Hilmersson, D. Ansari, R. Andersson, B. Andersson

**Affiliations:** Department of Surgery, Clinical Sciences Lund, Surgery, Lund University and Skåne University Hospital, 221 85 Lund, Sweden

**Keywords:** Distal cholangiocarcinoma, Pancreatic ductal adenocarcinoma, Thrombospondin-2, CA 19-9, Biomarker

## Abstract

**Purpose:**

Distal cholangiocarcinoma and pancreatic ductal adenocarcinoma are malignancies with poor prognoses that can be difficult to distinguish preoperatively. Thrombospondin-2 has been proposed as a novel diagnostic biomarker for early pancreatic ductal adenocarcinoma. The aim of the present study was to evaluate thrombospondin-2 as a diagnostic and prognostic biomarker in combination with current biomarker CA 19-9 for distal cholangiocarcinoma and pancreatic ductal adenocarcinoma.

**Methods:**

Thrombospondin-2 was measured in prospectively collected serum samples from patients who underwent surgery with a histopathological diagnosis of distal cholangiocarcinoma (*N* = 51), pancreatic ductal adenocarcinoma (*N* = 52) and benign pancreatic diseases (*N* = 27) as well as healthy blood donors (*N *= 52) using an enzyme-linked immunosorbent assay.

**Results:**

Thrombospondin-2 levels (ng/ml) were similar in distal cholangiocarcinoma 55 (41–77) and pancreatic ductal adenocarcinoma 48 (35–80) (*P* = 0.221). Thrombospondin-2 + CA 19-9 had an area under the curve of 0.92 (95% CI 0.88–0.97) in differentiating distal cholangiocarcinoma and pancreatic ductal adenocarcinoma from healthy donors which was superior to CA 19-9 alone (*P* < 0.001). The diagnostic value of adding thrombospondin-2 to CA 19-9 was larger in early disease stages. Thrombospondin-2 did not provide additional value to CA 19-9 in differentiating the benign disease group; however, heterogeneity was notable in the benign cohort. Three of five patients with autoimmune pancreatitis patients had greatly elevated thrombospondin-2 levels. Thrombospondin-2 levels had no correlation with prognoses.

**Conclusions:**

Serum thrombospondin-2 in combination with CA 19*-*9 has potential as a biomarker for distal cholangiocarcinoma and pancreatic cancer.

**Supplementary Information:**

The online version contains supplementary material available at 10.1007/s12094-021-02685-8.

## Introduction

Cholangiocarcinoma (CCA) constitutes diverse cancers originating from the biliary tree, accounting for approximately 3% of gastrointestinal cancers [1]. Many patients currently present with advanced disease and are unamenable to curative resection [2]. Distal cholangiocarcinoma (dCCA) is a subgroup that arises in the region of the common bile duct that traverses the pancreatic head. By definition, dCCA is located between the cystic duct insertion and the Ampulla of Vater [3]. Approximately 20–30% of CCAs are classified as dCCAas [2]. The clinical presentation between dCCA and pancreatic ductal adenocarcinoma (PDAC) of the pancreatic head is similar [4]. It is generally difficult to differentiate the two malignancies prior to surgical resection and histopathological evaluation [4]. Even after an adequate diagnostic workup, 5–13% of resections with a preoperative suspicion of pancreatic head malignancy are due to benign conditions [5].

There is currently only one biomarker in use for the diagnosis of both CCA and PDAC, namely serum carbohydrate antigen 19-9 (CA 19-9). Obstructive jaundice can cause CA 19-9 elevation in benign conditions [6]. In addition, patients with the Lewis a- b- genotype, representing 5–10% in Caucasian populations, are unable to express CA 19-9 [7]. Limited sensitivity and specificity, especially in early stages, means that the primary use of CA 19-9 is in monitoring disease progression [8].

Thrombospondin-2 (THBS2) is a member of the thrombospondin superfamily. It is a matricellular glycoprotein previously implicated in angiogenesis [9], extracellular matrix assembly [10] and apoptosis [11]. Recently, Kim et al. identified THBS2 as secreted from a pancreatic cancer precursor lesion model [12]. Further validation revealed THBS2 to be an excellent diagnostic biomarker compared to healthy donors (HDs) and patients with benign diseases (BDs), such as chronic pancreatitis and low*-*risk intraductal papillary mucinous neoplasms (IPMNs). In particular, THBS2 was able to accurately identify patients in early/resectable disease stages [12]. The usefulness of THBS2 as a diagnostic biomarker for PDAC in combination with other biomarkers has since been validated in other cohorts [13–15]. We recently observed THBS2 upregulated in both cancer cells and stromal cells in dCCA specimens using mass spectrometry and immunohistochemistry [16].

The purpose of the present study was to evaluate the expression of serum THBS2 in dCCA and assess its performance as a diagnostic biomarker for dCCA compared to cohorts with PDAC, BDs and HDs. Furthermore, subgroup analysis on the utility of THBS2 as a diagnostic biomarker across stage groups and the impact of obstructive jaundice was performed. Finally, the prognostic impact of serum THBS2 in dCCA and PDAC was evaluated.

## Materials and methods

### Study population

Blood samples were prospectively collected from patients who underwent pancreatic surgery at the Department of Surgery, Skåne University Hospital between 2012 and 2019. Samples were collected prior to surgery, and patients who received neoadjuvant treatment were excluded. For the present study, all consecutive patients who underwent surgery with curative intent and had a histopathological diagnosis of dCCA were identified (*N* = 52). One patient initially included in the dCCA cohort was removed from analysis due to a coding error. Patients from the same cohort who had a histopathological diagnosis of PDAC were matched 1:1 to the highest extent possible for the presence of lymph node metastases and T stage distribution within the dCCA cohort. The BD cohort included all patients who underwent pancreatic surgery but received a benign histopathological diagnosis during the inclusion period. Individuals with dysplastic changes above low-grade dysplasia present were excluded. The BD cohort (*N* = 27) consisted of patients with low*-*risk intraductal papillary mucinous neoplasm (IPMN) (*N* = 4), mucinous cystadenoma (*N* = 1), adenoma/serous cystadenoma (*N* = 11), and chronic pancreatitis/pseudocyst (*N* = 5), concomitant IPMN (*N* = 1) and autoimmune pancreatitis (*N* = 5). Samples from age- and sex 1:1-matched HDs were retrieved from the local blood donation centre. No power calculation was performed.

Demographic and clinicopathological data as well as preoperative total bilirubin levels (µmol/l) were retrospectively retrieved from medical charts. Staging was performed in accordance with the American Joint Committee on Cancer (AJCC) 7th edition until December 31, 2017, after which staging according to the 8th edition was used. To compare levels of THBS2 and CA 19-9 across stages, the amalgamated stage (I, II or III) regardless of AJCC version was used. In addition, to increase comparability across the study period and between dCCA and PDAC, a separate analysis was performed on the levels of THBS2 and CA 19-9 where the N stage was reclassified for all samples in accordance with the AJCC 8th edition which has identical N stage criteria for both dCCA and PDAC. Patients were monitored up to 3 years postoperatively for recurrence, and survival status was consequently censored at 3 years postoperatively and recorded July 6, 2020. The study is reported in accordance with the STARD guidelines where applicable [17].

### Serum sample collection

Blood samples were collected into serum separator tubes BD SST II Advance tubes (368,498, BD Vacutainer Systems, Franklin Lakes, NJ, USA). After a 30 min clotting time, the sera were separated by centrifugation at 2000 × *g* for 10 min at 25 °C. The sera were stored in aliquots at  – 80 °C until further analysis.

### Serum THBS2 and CA 19-9

The serum levels of human thrombospondin-2 were determined by quantitative sandwich enzyme-linked immunosorbent assay (ELISA) using a commercially available kit (DTSP20, R&D Systems, Minneapolis, MN, USA). The assay was performed according to the manufacturer’s instructions. All measurements were performed in technical duplicates. First or second thaw cycles were used. Samples were diluted four- to tenfold in calibrator diluent. Optical density was determined using a Multiskan GO microplate spectrophotometer (Thermo Fisher Scientific Oy, Vantaa, Finland) at 450 nm and 540 nm to correct for optical imperfections. Sample concentrations were determined from a standard curve of the positive controls included in the kit. A four-parameter logistic regression standard curve was generated using MyAssays online data analysis tool (MyAssays Ltd., accessed June 2020, http://www.myassays.com/four-parameter-logistic-curve.assay). Four samples had a higher concentration than the range of the standard curve at a tenfold dilution, and values were extrapolated by extending the standard curve.

Serum levels of CA 19-9 were measured in a clinical laboratory (Department of Clinical Chemistry and Pharmacology, University and Regional Laboratories Region Skåne, Sweden) using a clinically accredited immunoassay (Ref.11776193 122 Cobas/Roche).

### Statistical analysis

Statistical analysis was performed using Stata MP statistical package version 14.2 for Mac OS X (Stata corporation LP, College Station, TX) and GraphPad Prism version 8.4.3 for Mac OS (GraphPad Software, San Diego, California, USA). Values are given as medians with interquartile ranges (IQRs). For categorical values absolute numbers and the distribution in percentages on available data are given. The Mann–Whitney *U* or Kruskal–Wallis H test was used to compare medians of continuous variables. Contingency tables with x^2^ or Fisher’s exact test were used for categorical variables as appropriate.

CA 19-9 was dichotomised at 35 kU/l and bilirubin at 25 µmol/l in accordance with the clinical upper limit of normal. The cutoff 42 ng/ml for THBS2 expression in PDAC was suggested by Kim et al. [12] and used in several subsequent studies evaluating THBS2 as a diagnostic biomarker [14, 15, 18]. The cutoff was selected to represent a false-positive rate of 1% in HDs and translated to a sensitivity of 52% and a specificity of 99% for THBS2 when comparing PDAC vs HDs [12]. In the present study, the previously used cutoff at 42 ng/ml corresponded approximately to the 92nd percentile in the HDs. Due to unknown technical variation, the average THBS2 value in the HDs was 31 in the present study compared to 17 in the study by Kim et al. [12] As such, we chose to adopt the cutoff of 42 ng/ml directly and also selected the cutoff 51 ng/ml which yielded comparable diagnostic performance in the combined dCCA and PDAC cohort as previously presented for 42 ng/ml.

Univariable and multivariable logistic regressions were used to evaluate the diagnostic performance of individual markers and the combination of markers. All significance tests comparing the area under the curve (AUC) between models were performed against CA 19-9 alone. Survival was estimated using the Kaplan–Meier method and compared using the log-rank test.

## Results

### Expression of serum THBS2 and CA 19-9

**Fig. 1:**
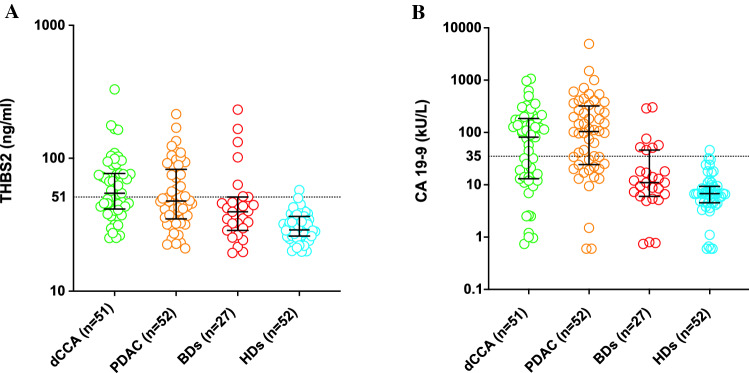
Scatter plots of THBS2 (**A**) and CA 19-9 (**B**) concentrations in the dCCA, PDAC, BD and HD cohorts. Cutoffs at 51 ng/ml for THBS2 and 35 kU/l for CA 19-9 are highlighted. A logarithmic scale is presented

The study included 51 patients with dCCA, 52 patients with PDAC, 27 patients with BDs and 52 HDs. The clinicopathological data and ELISA measurements are presented in Table 1. There was no significant difference in the THBS2 value between dCCA and PDAC (*P* = 0.221). THBS2 values were significantly higher in dCCA than in BDs (*P* = 0.005) and HDs (*P* < 0.001). THBS2 values were not significantly higher in PDAC than in BDs (*P* = 0.069) but were significantly higher than in HDs (*P* < 0.001). There was no significant difference in CA 19-9 levels between dCCA and PDAC (*P* = 0.096). CA 19-9 was significantly higher in dCCA than in BDs (*P* = 0.001) and HDs (*P* < 0.001). Similarly, CA 19-9 was higher in PDAC than in BDs (*P* < 0.001) and HDs (*P* < 0.001). The distribution of THBS2 and CA 19-9 values is presented as a scatterplot in Fig. 1.

**Table 1:** Clinicopathological data in the cohorts evaluated for serum THBS2 expression

	dCCA (*N* = 51)	PDAC (*N* = 52)	BDs (*N* = 27)	HDs (*N* = 52)
Variable				
Age	69 (61–76)	69 (64–73)	66 (58–72)	66 (63–68)
Sex				
Female	28 (55)	26 (50)	15 (56)	28 (54)
BMI (kg/m^2^)	27 (23–28)	25 (23–29)	26 (23–28)	
Diabetes				
Present	10 (20)	20 (38)	6 (22)	
THBS2 (ng/ml)	55 (41–77)	48 (35–80)	40 (29–51)	29 (26–36)
CA 19-9 (kU/l)	81 (13–184)	104 (25–303)	11 (6–46)	7 (5–9)
Elevated (≥ 35 kU/l)	29 (57)	35 (67)	7 (26)	1 (2)
Bilirubin (µmol/l)	18 (11–41)	15 (6–40)	10 (6–13)	
Elevated (> 25 µmol/l)	20 (41)	18 (35)	2 (7)	
AJCC stage 7th + 8th				
I	5 (10)	5 (10)		
II	37 (73)	47 (90)		
III	9 (18)	0		
AJCC 7th				
I	4 (13)	1 (2)		
II	25 (83)	40 (98)		
III	1 (3)	0		
AJCC 8th				
I	1 (5)	4 (36)		
II	12 (57)	7 (64)		
III	8 (38)	0		
AJCC 8th N stage				
N0	20 (39)	17 (33)		
N1	14 (27)	15 (29)		
N2	17 (33)	20 (38)		
R1 resection				
Present	26 (51)	39 (75)		

*AJCC* American Joint Committee on cancer, *BDs* benign diseases, *BMI* body mass index, *CA 19-9* carbohydrate antigen 19-9, *dCCA* distal cholangiocarcinoma, *HDs* healthy donors, *PDAC* pancreatic ductal adenocarcinoma, *R1* non-radical resection, *THBS2* thrombospondin-2

There was no significant difference in THBS2 levels in dCCA or PDAC when stratified by amalgamated AJCC stage or AJCC 8th edition N stage. This was in contrast to CA 19-9 levels, which were significantly higher across AJCC stages in dCCA (*P* = 0.019) and AJCC 8th edition N stages in PDAC (*P* = 0.009) (Supplementary 1).

### Diagnostic performance of THBS2 and CA 19-9 in dCCA and PDAC compared with HDs

**Fig. 2:**
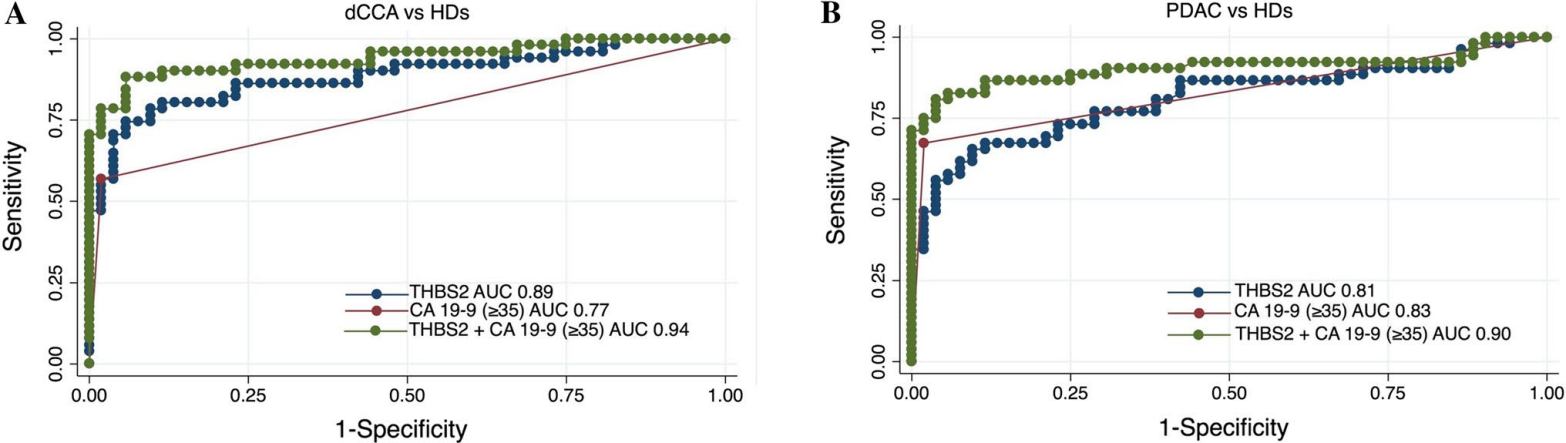
ROC curves for THBS2, CA 19-9 and the combined THBS2 + CA 19-9 in dCCA vs HDs (**A**), PDAC vs HDs (**B**). Abbreviations: *CA 19*-*9* carbohydrate antigen 19-9, *dCCA* distal cholangiocarcinoma, *HDs* healthy donors, *PDAC* pancreatic ductal adenocarcinoma, *THBS2* thrombospondin-2

The discriminatory power (i.e. AUC) for THBS2 in dCCA versus HDs was 0.89 (95% CI 0.82–0.95), and for CA 19-9 (≥ 35), it was 0.77 (95% CI 0.70–0.85). By combining the two markers, the discriminatory power was significantly improved, with an AUC of 0.94 (95% CI 0.89–0.99) (*P* < 0.001) (Fig. 2) (Supplementary 2). The discriminatory power for THBS2 in PDAC versus HDs was 0.81 (95% CI 0.72–0.90) and for CA 19-9 (≥ 35) 0.83 (95% CI 0.76–0.89); the combination of markers had an AUC of 0.90 (95% CI 0.84–0.97, *P* = 0.017) (Fig. 2) (Supplementary 2). By combining dCCA and PDAC as an endpoint, THBS2 had an AUC of 0.85 (95% CI 0.79–0.91), CA 19-9 (≥ 35) alone had an AUC of 0.80 (95% CI 0.75–0.85), and the combination had an enhanced AUC of 0.92 (95% CI 0.88–0.97, *P* < 0.001).

When stratified by amalgamated AJCC stage and AJCC 8th edition N stage, the addition of THBS2 to CA 19-9 seemed to provide the greatest diagnostic improvement in the early stages of dCCA and PDAC. In dCCA, THBS2 + CA 19-9 (≥ 35) had an AUC of 0.87 compared to 0.69 with CA 19-9 (≥ 35) alone for N0 patients (Supplementary 2). In PDAC, THBS2 + CA 19-9 (≥ 35) had an AUC of 0.81 compared to 0.70 with CA 19-9 (≥ 35) alone in N0 patients (Supplementary 2).

The cutoff 51 ng/ml for THBS2, provided 50% sensitivity and 98% specificity when comparing dCCA + PDAC versus HDs which was comparable to the previously presented diagnostic performance of THBS2 at 42 ng/ml [12]. Combining THBS2 (≥ 51) and CA 19-9 (≥ 35), we observed a sensitivity of 79% and a specificity of 96% when comparing dCCA + PDAC versus HDs. This represented a substantial improvement compared to the sensitivity of 62% and specificity of 98% for CA 19-9 (≥ 35) alone. Diagnostic performance with dichotomisation at 42 ng/ml and 51 ng/ml in the different cohorts is presented in Supplementary 3. Notably, THBS2 (≥ 51) identified 11/22 (50%) of all CA 19-9-negative dCCA patients and 6/17 (35%) of all CA 19-9-negative PDAC patients, highlighting its additive value.

### Diagnostic performance of THBS2 and CA 19-9 in dCCA and PDAC compared with BDs

**Fig. 3:**
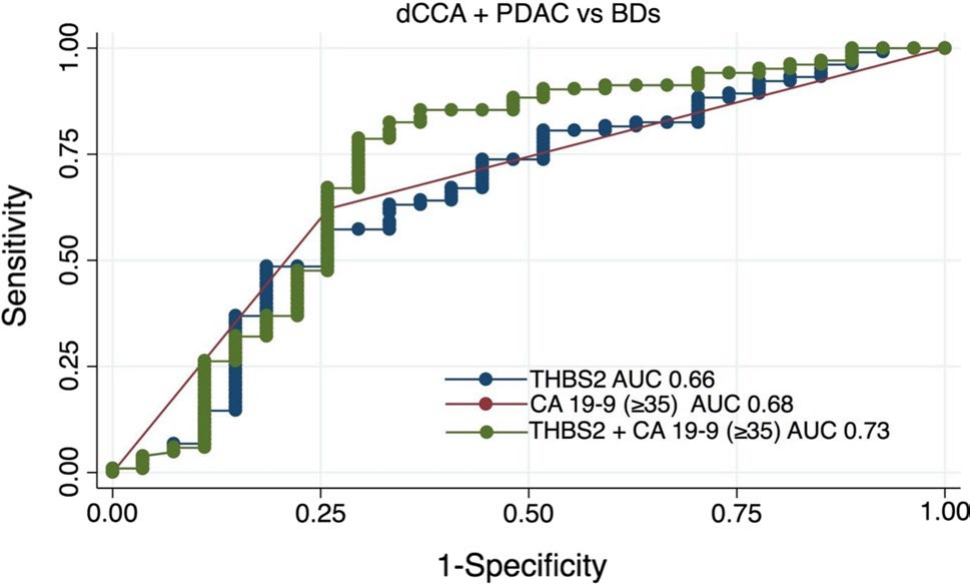
ROC curves for THBS2, CA 19-9 and THBS2 + CA 19-9 for the dCCA + PDAC cohort vs BDs. Abbreviations: *BDs* benign diseases, *CA 19*-*9* carbohydrate antigen 19-9, *dCCA* distal cholangiocarcinoma, *PDAC* pancreatic ductal adenocarcinoma, *THBS2* thrombospondin-2

The AUC for THBS2 in the combined endpoint of dCCA and PDAC versus BDs was 0.66 (95% CI 0.54–0.78) and for CA 19-9 (≥ 35) was 0.68 (95% CI 0.58–0.78). The combination of THBS2 and CA 19-9 (≥ 35) had an AUC of 0.73 (95% CI 0.60–0.86), which was not significantly better than that off CA 19-9 (≥ 35) alone (*P* = 0.166) (Fig. 3). Applying the cutoff (≥ 51) for THBS2, a sensitivity of 78% with a specificity of 67% was seen for THBS2 + CA 19-9 (≥ 35) (Supplementary 3).

In a secondary analysis, the BD cohort was grouped by diagnosis. The majority of BD samples had THBS2 values that largely overlapped with distribution in the HDs; however, four outliers consisting of three patients with autoimmune pancreatitis and one with IPMN and chronic pancreatitis had substantially higher values (Supplementary 4).

Elevated preoperative bilirubin levels were present in 20 (41%) patients with dCCA, 18 (35%) with PDAC and 2 (7%) with BD (Table 1). Median THBS2 values were significantly higher in patients with elevated bilirubin 75 (57–103) than in patients with normal bilirubin 42 (32–51) (*P* < 0.001). Median CA 19-9 values were not significantly different between patients with elevated bilirubin 101 (14–250) kU/l compared to patients with normal bilirubin 38 (12–147) kU/l (*P* = 0.184). In dCCA, THBS2 (≥ 51) identified four (31%) nonjaundiced, CA 19-9-negative patients. In PDAC, THBS2 (≥ 51) identified one (9%) nonjaundiced CA 19-9-negative patient.

### Prognostic role of THBS2 in dCCA and PDAC

Elevated THBS2 (≥ 51) was not associated with DFS (*P* = 0.846) or OS (P = 0.798) in dCCA. Similarly, no association with DFS (*P* = 0.932) or OS (*P* = 0.537) was seen in PDAC. In addition, no significant association with either DFS or OS was seen when THBS2 was dichotomised at the median expression value in dCCA and PDAC (data not shown).

## Discussion

The utility of THBS2 as a diagnostic biomarker for early-stage PDAC in combination with CA 19-9 and other markers has been previously demonstrated. In the present study, THBS2 + CA*-*19 (≥ 35) had an AUC of 0.9 when comparing PDAC versus HDs, which is in line with previous results [12–14, 18]. Neither THBS2 nor CA 19-9 has shown value in the prediagnostic setting [19]. THBS2 levels and diagnostic performance were similar between dCCA and PDAC, suggesting that THBS2 could also be used in the diagnosis of dCCA. This finding is in line with results by Large et al. [18]. The frequent dysregulation of THBS2 in several cancers [20] implies that it is not a specific marker for PDAC or dCCA, and it has been proposed as a diagnostic marker in colorectal cancer [21], hepatocellular cancer [22] and lung cancer [23]. Additional research is needed to elucidate the expression pattern of THBS2 in other subtypes of CCA and across carcinomas of different origins. Additionally, most studies on PDAC focus on biomarker panels to improve diagnostic precision [24]. Although there have been some promising results combining THBS2, CA 19-9 and cell-free DNA [14], the possibility to improve the diagnostic precision of THBS2 and CA 19-9 using additional biomarkers remains to be elucidated.

In the present study, THBS2 did not contribute diagnostic information compared to CA 19-9 alone when comparing dCCA or PDAC versus BDs. This result is similar to the study by Large et al. where dCCA and PDAC were compared to benign controls with chronic pancreatitis, cholangitis and choledocholithiasis, and the addition of THBS2 did not improve the performance of CA 19-9 alone [18]. However, the majority of previous studies have generally found THBS2 to reliably discriminate PDAC from benign pancreatic conditions such as chronic pancreatitis [12–14], IPMN [12, 14, 15] and patients with a family history of PDAC undergoing screening [13]. The reason behind the discrepancy could be in the selection of benign controls. The present control group consisted of patients who were considered to have a high risk of malignancy meriting surgical intervention and with histopathologically confirmed diagnoses. As such, they represent a realistic cohort of clinically misdiagnosed patients. However, elevated THBS2 levels in the BDs were diagnosis- and cutoff- dependent, with autoimmune pancreatitis showing substantially elevated THBS2 levels. Further evaluation in larger cohorts of different benign conditions and cutoff optimisation for this clinical situation needs to be performed if THBS2 is to be used as a biomarker. The finding that higher THBS2 levels were observed in jaundiced patients is consistent with previous results [12, 18]. Interestingly, Large et al. found that although THBS2 did not improve the performance compared to CA 19-9 in identifying dCCA + PDAC versus BDs, it did have diagnostic value in jaundiced patients, a hypothesis we could not evaluate since we had too few benign controls with jaundice. In the present study, preoperative bilirubin values were obtained preoperatively from patient charts.

We did not find any correlation between THBS2 expression and stage or survival in dCCA or PDAC. Previously, Peng et al. found THBS2 expression to be associated with poor survival in a large cohort of PDAC patients of Asian origin. Differences in sample size, patient selection or ethnic differences could contribute to the divergent results.

Some limitations to the present study can be noted. The sample size which was based on availability of samples rather than a power calculation is small, which limits the statistical power especially in the subgroup analysis. In particular, the BD cohort was small, and very few patients had individual benign conditions limiting conclusion that can be drawn. We did not include any high-risk cohorts such as newly diagnosed diabetes or genetic risk groups where asymptomatic screening could be considered. The BD cohort only consisted of pancreatic conditions and no benign biliary conditions were evaluated. All groups were well matched with regard to sex but the dCCA and PDAC cohorts were slightly older than the BDs and HDs. The matching of dCCA and PDAC patients was imperfect, with more PDAC patients at higher N stages than dCCA patients. The measurement of preoperative bilirubin levels was retrospectively acquired from patient charts. During the study period, many patients at our institution underwent preoperative biliary drainage prior to surgery, and as such, bilirubin levels do not represent the initial diagnostic workup situation. In addition, bilirubin measurements are influenced by time from drainage and are not routinely obtained at the same timepoint as the THBS2 and CA 19-9 samples. Although the diagnostic performance of THBS2 was similar in the present study as in previous studies using the same ELISA, we had higher measurements in the HD group, likely representing technical variation. Assay optimisation and cutoff standardisation are required prior to the clinical use of THBS2 as a diagnostic biomarker.

In conclusion, we found that serum THBS2 in combination with CA 19-9 has potential as a biomarker combination for dCCA and PDAC. Importantly, THBS2 helped identify early disease stages where curative treatment is possible. Further research is needed to understand the expression of THBS2 in different malignant and benign conditions as well as assay development prior to clinical use.

## Supplementary Information

Below is the link to the electronic supplementary material.

Supplementary file1 (DOCX 19 KB)

Supplementary file2 (DOCX 16 KB)

Supplementary file3 (DOCX 15 KB)

Supplementary file4 (DOCX 79 KB)

## Data Availability

The datasets generated during the present study are available from the corresponding author upon reasonable request.
