# Supplementary material for: Thrombospondin-2 as a diagnostic biomarker for distal cholangiocarcinoma and pancreatic ductal adenocarcinoma
Source: Clin Transl Oncol. 2021 Jul 28;24(2):297–304. doi: 10.1007/s12094-021-02685-8 (PMC8794913; doi:10.1007/s12094-021-02685-8)
Supplement: Supplementary file 1 — Supplementary file1 (DOCX 19 KB) [file 12094_2021_2685_MOESM1_ESM.docx]

Thrombospondin-2 as a diagnostic biomarker for distal cholangiocarcinoma and pancreatic ductal adenocarcinoma

Clinical and Translational Oncology

Johannes Byrling, M.D. Katarzyna Said Hilmersson, Daniel Ansari, M.D., Ph.D. Roland Andersson, M.D., Ph.D. Bodil Andersson, M.D., Ph.D.

Department of Clinical Sciences Lund, Surgery, Lund University and Skåne University Hospital, Lund, Sweden

Correspondence to:

Bodil Andersson, M.D., Ph.D.

Department of Surgery, Clinical Sciences Lund

Lund University and Skåne University Hospital, Lund

SE-221 85 Lund, Sweden

Tel: + 46 46 17 27 57

E-mail: bodil.andersson@med.lu.se

**Supplementary 1.**  THBS2 and CA 19-9 ELISA levels stratified by amalgamated AJCC stage (A) and N stage from AJCC^8th^ edition (B)

**A**

|  |  | |  |  |  |  |  |  |
| --- | --- | --- | --- | --- | --- | --- | --- | --- |
|  | AJCC Stage I | AJCC Stage II | AJCC Stage III | P-value |  |  |  |  |
| **dCCA** |  |  |  |  |  |  |  |  |
| N | 5 | 37 | 9 |  |  |  |  |  |
| THBS2 (ng/ml) | 62 (43–89) | 51 (41–76) | 70 (43–99) | 0.670 |  |  |  |  |
| CA 19-9 (kU/L) | 16 (7–104) | 57 (12–175) | 198 (127–494) | 0.019 |  |  |  |  |
| **PDAC** |  |  |  |  |  |  |  |  |
| N | 5 | 47 |  |  |  |  |  |  |
| THBS2 (ng/ml) | 35 (32–92) | 48 (37–75) |  | 0.609 |  |  |  |  |
| CA 19-9 (kU/L) | 22 (14–145) | 106 (30–334) |  | 0.313 |  |  |  |  |

**B**

|  |  | |  |  |  |  |  |  |
| --- | --- | --- | --- | --- | --- | --- | --- | --- |
|  | AJCC^8th^ N0 | AJCC^8th^ N1 | AJCC^8th^ N2 | P-value |  |  |  |  |
| **dCCA** |  |  |  |  |  |  |  |  |
| N | 20 | 14 | 17 |  |  |  |  |  |
| THBS2 (ng/ml) | 60 (42–90) | 49 (40–76) | 59 (45–75) | 0.831 |  |  |  |  |
| CA 19-9 (kU/L) | 15 (10–116) | 38 (14–212) | 127 (81–198) | 0.062 |  |  |  |  |
| **PDAC** |  |  |  |  |  |  |  |  |
| N | 17 | 15 | 20 |  |  |  |  |  |
| THBS2 (ng/ml) | 41 (31–58) | 50 (42–75) | 51 (35–95) | 0.281 |  |  |  |  |
| CA 19-9 (kU/L) | 34 (20–145) | 41 (17–361) | 215 (109–411) | 0.009 |  |  |  |  |

Abbreviations: AJCC; American Joint Committee on cancer. CA 19-9; Carbohydrate antigen 19-9. CI; confidence interval. dCCA; distal cholangiocarcinoma. N stage; nodal stage. PDAC; pancreatic ductal adenocarcinoma. THBS2; thrombospondin-2.
