# Supplementary material for: Thrombospondin-2 as a diagnostic biomarker for distal cholangiocarcinoma and pancreatic ductal adenocarcinoma
Source: Clin Transl Oncol. 2021 Jul 28;24(2):297–304. doi: 10.1007/s12094-021-02685-8 (PMC8794913; doi:10.1007/s12094-021-02685-8)
Supplement: Supplementary file 2 — Supplementary file2 (DOCX 16 KB) [file 12094_2021_2685_MOESM2_ESM.docx]

Thrombospondin-2 as a diagnostic biomarker for distal cholangiocarcinoma and pancreatic ductal adenocarcinoma

Clinical and Translational Oncology

Johannes Byrling, M.D. Katarzyna Said Hilmersson, Daniel Ansari, M.D., Ph.D. Roland Andersson, M.D., Ph.D. Bodil Andersson, M.D., Ph.D.

Department of Clinical Sciences Lund, Surgery, Lund University and Skåne University Hospital, Lund, Sweden

Correspondence to:

Bodil Andersson, M.D., Ph.D.

Department of Surgery, Clinical Sciences Lund

Lund University and Skåne University Hospital, Lund

SE-221 85 Lund, Sweden

Tel: + 46 46 17 27 57

E-mail: bodil.andersson@med.lu.se

**Supplementary 2.** AUC calculations* of ELISA results stratified by amalgamated AJCC ^7th+8th^ stage and N stage from AJCC^8th^ edition.

|  |  | *THBS2* | | CA 19-9 (≥35) | | THBS2+CA 19-9 (≥35) | |  |
| --- | --- | --- | --- | --- | --- | --- | --- | --- |
|  | ***N*** | ***AUC*** | ***95% CI*** | ***AUC*** | ***95% CI*** | ***AUC*** | ***95% CI*** | **P-value** |
| **dCCA vs HDs** | 51 | 0.89 | 0.82–0.95 | 0.77 | 0.70–0.85 | 0.94 | 0.89–0.99 | <0.001 |
| AJCC Stage I | 5 | 0.84 | 0.56–1.00 | 0.69 | 0.45–0.93 | 0.85 | 0.56–1.00 | 0.290 |
| AJCC Stage II | 37 | 0.88 | 0.81–0.96 | 0.75 | 0.66–0.83 | 0.94 | 0.89–1.00 | <0.001 |
| AJCC Stage III | 9 | 0.92 | 0.81–1.00 | 0.93 | 0.82–1.00 | 0.97 | 0.92–1.00 | 0.211 |
| AJCC^8th^ N0 | 20 | 0.87 | 0.77–0.98 | 0.69 | 0.58–0.8 | 0.87 | 0.76–0.98 | 0.003 |
| AJCC^8th^ N1 | 14 | 0.87 | 0.74–1.00 | 0.74 | 0.60–0.88 | 0.99 | 0.97–1.00 | <0.001 |
| AJCC^8th^ N2 | 17 | 0.92 | 0.82–1.00 | 0.90 | 0.81–1.00 | 0.98 | 0.96–1.00 | 0.048 |
| **PDAC vs HDs** | 52 | 0.81 | 0.72–0.90 | 0.83 | 0.76–0.89 | 0.90 | 0.84–0.97 | 0.017 |
| AJCC Stage I | 5 | 0.68 | 0.37–1.00 | 0.69 | 0.45–0.93 | 0.76 | 0.42–1.00 | 0.623 |
| AJCC Stage II | 42 | 0.82 | 0.74–0.91 | 0.84 | 0.77–0.91 | 0.92 | 0.85–0.99 | 0.017 |
| AJCC^8th^ N0 | 17 | 0.71 | 0.54–0.88 | 0.70 | 0.57–0.82 | 0.81 | 0.64–0.97 | 0.141 |
| AJCC^8th^ N1 | 15 | 0.90 | 0.78–1.00 | 0.79 | 0.66–0.92 | 0.92 | 0.80–1.00 | 0.059 |
| AJCC^8th^ N2 | 20 | 0.83 | 0.7–0.95 | 0.97 | 0.91–1.00 | 0.98 | 0.93–1.00 | 0.204 |

Abbreviations: AJCC; American Joint Committee on Cancer. AUC; area under curve. CA 19-9; Carbohydrate antigen 19-9. CI; confidence interval. dCCA; distal cholangiocarcinoma. HDs; healthy donors. N stage; nodal stage. PDAC; pancreatic ductal adenocarcinoma. THBS2; thrombospondin-2.

*P-value calculated from the logistic regression comparing AUC from THBS2+ CA 19-9 (≥35) to the AUC for CA 19-9 (≥35) alone.
