# Supplementary material for: Thrombospondin-2 as a diagnostic biomarker for distal cholangiocarcinoma and pancreatic ductal adenocarcinoma
Source: Clin Transl Oncol. 2021 Jul 28;24(2):297–304. doi: 10.1007/s12094-021-02685-8 (PMC8794913; doi:10.1007/s12094-021-02685-8)
Supplement: Supplementary file 3 — Supplementary file3 (DOCX 15 KB) [file 12094_2021_2685_MOESM3_ESM.docx]

Thrombospondin-2 as a diagnostic biomarker for distal cholangiocarcinoma and pancreatic ductal adenocarcinoma

Clinical and Translational Oncology

Johannes Byrling, M.D. Katarzyna Said Hilmersson, Daniel Ansari, M.D., Ph.D. Roland Andersson, M.D., Ph.D. Bodil Andersson, M.D., Ph.D.

Department of Clinical Sciences Lund, Surgery, Lund University and Skåne University Hospital, Lund, Sweden

Correspondence to:

Bodil Andersson, M.D., Ph.D.

Department of Surgery, Clinical Sciences Lund

Lund University and Skåne University Hospital, Lund

SE-221 85 Lund, Sweden

Tel: + 46 46 17 27 57

E-mail: bodil.andersson@med.lu.se

**Supplementary 3.** Diagnostic performance of THBS2 and CA 19-9 at selected cutoffs.

|  | Sensitivity (%) | Specificity (%) |
| --- | --- | --- |
| **dCCA + PDAC vs HDs** |  |  |
| THBS2 (≥51) | 49.5 | 98.1 |
| THBS2 (≥42) | 70.0 | 92.3 |
| CA 19-9 (≥35) | 62.1 | 98.1 |
| THBS2 (≥51) + CA 19-9 (≥35) | 78.6 | 96.2 |
| THBS2 (≥42) + CA 19-9 (≥35) | 85.4 | 90.4 |
| **dCCA vs HDs** |  |  |
| THBS2 (≥51) | 54.9 | 98.1 |
| THBS2 (≥42) | 74.5 | 92.3 |
| CA 19-9 (≥35) | 56.9 | 98.1 |
| THBS2 (≥51) + CA 19-9 (≥35) | 78.4 | 96.2 |
| THBS2 (≥42) + CA 19-9 (≥35) | 88.2 | 90.4 |
| **PDAC vs HDs** |  |  |
| THBS2 (≥51) | 44.2 | 98.1 |
| THBS2 (≥42) | 59.6 | 92.3 |
| CA 19-9 (≥35) | 67.3 | 98.1 |
| THBS2 (≥51) + CA 19-9 (≥35) | 78.8 | 96.2 |
| THBS2 (≥42) + CA 19-9 (≥35) | 82.7 | 90.4 |
| **dCCA + PDAC vs BDs** |  |  |
| THBS2 (≥51) | 49.1 | 74.1 |
| THBS2 (≥42) | 70.0 | 55.6 |
| CA 19-9 (≥35) | 62.1 | 74.1 |
| THBS2 (≥51) + CA 19-9 (≥35) | 78.6 | 66.7 |
| THBS2 (≥42) + CA 19-9 (≥35) | 85.4 | 51.9 |
| **dCCA vs BDs** |  |  |
| THBS2 (≥51) | 54.9 | 74.1 |
| THBS2 (≥42) | 74.5 | 55.6 |
| CA 19-9 (≥35) | 56.9 | 74.1 |
| THBS2 (≥51) + CA 19-9 (≥35) | 78.4 | 66.7 |
| THBS2 (≥42) + CA 19-9 (≥35) | 88.2 | 51.9 |
| **PDAC vs BDs** |  |  |
| THBS2 (≥51) | 44.2 | 74.1 |
| THBS2 (≥42) | 59.6 | 55.6 |
| CA 19-9 (≥35) | 67.3 | 74.1 |
| THBS2 (≥51) + CA 19-9 (≥35) | 78.8 | 66.7 |
| THBS2 (≥42) + CA 19-9 (≥35) | 82.7 | 51.9 |

Abbreviations: AUC; area under curve. BDs; benign diseases. CA 19-9; Carbohydrate antigen 19-9. CI; confidence interval. dCCA; distal cholangiocarcinoma. HDs; healthy donors. PDAC; pancreatic ductal adenocarcinoma. THBS2; thrombospondin-2.
