# Supplementary material for: Thrombospondin-2 as a diagnostic biomarker for distal cholangiocarcinoma and pancreatic ductal adenocarcinoma
Source: Clin Transl Oncol. 2021 Jul 28;24(2):297–304. doi: 10.1007/s12094-021-02685-8 (PMC8794913; doi:10.1007/s12094-021-02685-8)
Supplement: Supplementary file 4 — Supplementary file4 (DOCX 79 KB) [file 12094_2021_2685_MOESM4_ESM.docx]

Thrombospondin-2 as a diagnostic biomarker for distal cholangiocarcinoma and pancreatic ductal adenocarcinoma

Clinical and Translational Oncology

Johannes Byrling, M.D. Katarzyna Said Hilmersson, Daniel Ansari, M.D., Ph.D. Roland Andersson, M.D., Ph.D. Bodil Andersson, M.D., Ph.D.

Department of Clinical Sciences Lund, Surgery, Lund University and Skåne University Hospital, Lund, Sweden

Correspondence to:

Bodil Andersson, M.D., Ph.D.

Department of Surgery, Clinical Sciences Lund

Lund University and Skåne University Hospital, Lund

SE-221 85 Lund, Sweden

Tel: + 46 46 17 27 57

E-mail: bodil.andersson@med.lu.se

**Supplementary 4.** Scatter plot of THBS2 in dCCA, PDAC, HDs and BDs subgrouped by diagnosis. Cut-off levels of 51 ng/ml for THBS2 A logarithmic scale is presented.
